# Molecular mechanisms of underlying genetic factors and associated mutations for drug resistance in *Mycobacterium tuberculosis*

**DOI:** 10.1080/22221751.2020.1785334

**Published:** 2020-07-16

**Authors:** Shasank S. Swain, Divakar Sharma, Tahziba Hussain, Sanghamitra Pati

**Affiliations:** a Division of Microbiology and NCDs, ICMR-Regional Medical Research Centre, Bhubaneswar, India; b CRF, Mass Spectrometry Laboratory, Kusuma School of Biological Sciences (KSBS), Indian Institute of Technology-Delhi (IIT-D), Delhi, India; c Division of Public Health and Research, ICMR-Regional Medical Research Centre, Bhubaneswar, India

**Keywords:** Multi-drug-resistant tuberculosis, drug resistance mechanisms, anti-tubercular natural products, genetic factors, mutations

## Abstract

Nowadays, drug-resistant tuberculosis (DR-TB) and co-infected tuberculosis (CI-TB) strains are the leading cause for the enhancement of long-term morbidity and unpredicted mortality rates from this ghoulish acid fast-bacterium infection, globally. Unfortunately, the lack of/ample lethargic towards the development of compelling anti-TB regimens with a large-scale prevalence rate is a great challenge towards control of the pandemic situation. Indeed, the recent improvement in genomic studies for early diagnosis and understanding the mechanisms of drug resistance, as well as the identification of newer drug targets is quite remarkable and promising. Mainly, identification of such genetic factors, chromosomal mutations and associated pathways gives new ray of hope in current anti-TB drug discovery. This focused review provides molecular insights into the updated drug resistance mechanisms with encoded bacilli genetic factors as a novel target and potential source of development with screened-out newer anti-TB agents towards the control of MDR-TB soon.

## Introduction

*Mycobacterium tuberculosis* (Mtb) is a grievous pathogenic organism, which has infected about one-third of the world’s population globally [1,2]. According to the World Health Organization (WHO) statistics, each year there were ∼ 8–10 million new tuberculosis (TB) cases continuously recorded [1,2]. African and Asian countries are suffering more than the Western Pacific region, so overall, 7–8 million new Mtb cases have emerged from African and Asian countries [1]. In 2015, 60% of new Mtb cases had been recorded from India, Indonesia, China, Nigeria, Pakistan, and South Africa [1]. The failure of first-line drugs (isoniazid or INH, rifampicin or RIF, ethambutol or EMB, pyrazinamide or PZA and streptomycin or STR), second-line drugs (moxifloxacin or MXF, kanamycin or KAN, capreomycin or CAP, ethionamide or ETH, para-aminosalicylic acid or PAS and cycloserine or DCS) and ineffectiveness of Directly Observed Treatment Short (DOTS) are the leading cause for the emergence of multi-drug resistant (MDR), extensively drug-resistant (XDR), extremely drug-resistant (XXDR) and ghoulish totally drug-resistant (TDR) strains, worldwide [3]. Apart from that the increased rate of TB-HIV (Human Immunodeficiency Virus) co-infection is another issue leading to the pathetic situation towards the control of MDR-TB strain. In 2005, WHO reported ∼ 0.4 million death by TB-HIV co-infections [1,2].

In 1944, after the discovery of STR, TB treatment had been possible which created a revolution in anti-TB drug development. After that, there were several new classes of drugs introduced for treatment and recorded valid for a specific time against TB [3,4]. But nowadays, all mono and combination therapies, including DOTS and DOTS-Plus, are ineffective against the MDR-TB strains or bad bugs. Thus, TB still remains as one of the leading infectious diseases worldwide. Every year, it has reported 100,000 or 3.9% new cases of RIF-resistant TB from total 480,000 MDR-TB cases. Nowadays, data of XDR-TB cases from 117 countries suggested that around 9.5% MDR-TB changed to XDR TB [2]. Approximately, 50%–70% TB cases are curable by current combinatorial chemotherapy, but it is not enough due to the spontaneous addition of new MDR-TB cases gradually. In comparison with other bacterial diseases, anti-TB drug resistance occurs just after the newly introduced chemotherapeutic agent. On the other hand, MDR-TB treatment rate is also expensive and time-consuming [3,4]. Currently used anti-TB drugs are not potential for killing the dormant and intracellular forms of Mtb. Thus, the present drug resistance situation gives a direct indication of searching the alternate and active anti-TB agent.

**Figure 1.**
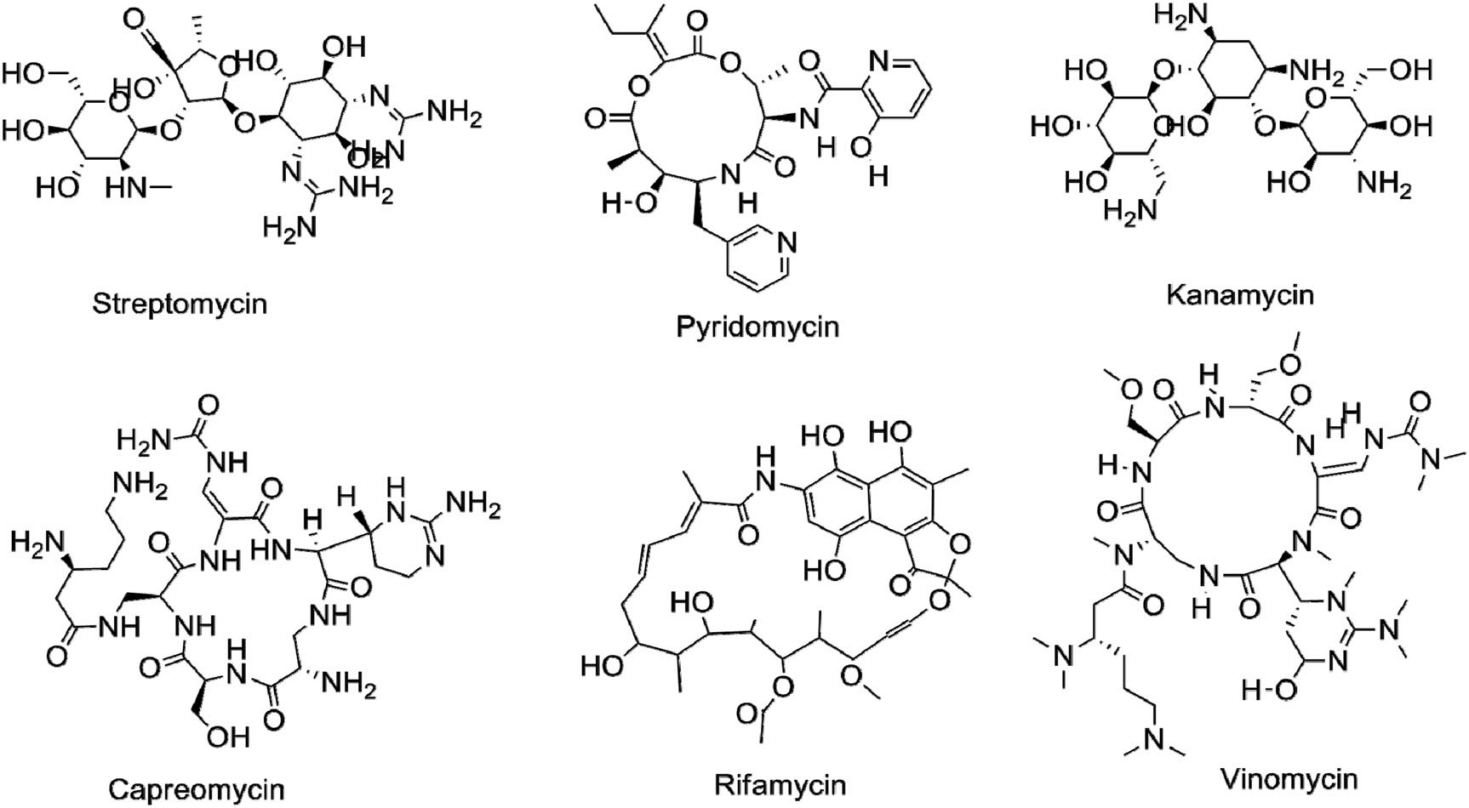
Successful anti-TB drugs from natural sources.

Since the last two decades, there has been a lot of progress to extract and identify the potent antimycobacterial chemicals/compounds from natural sources [5,6]. There are a lot of drugs discovered from the natural source such as STR and RIF [Figure 1]. Overall, natural sources are the main parental sources for the development of any semi-synthetic drug (RIF is a semi-synthetic-modified drug from rifamycin) [7,8]. WHO encourages and facilitates in national, regional and global level towards searching for new therapeutic agents for the eradication of TB in 2030. However, from the current status of anti-TB drug development record, WHO has changed their slogan “Stop TB to End TB.” With advancement in drug development techniques and instrumentation, there are several potential drug candidates developed with their novel mechanism of action including time-saving assays and ideal chemical isolation procedures compared to the previous drug discovery methods [6–9]. Therefore, in this timeline review, we have discussed updated information on drug-resistant mechanisms of Mtb.

## Drug-resistant mechanisms of Mtb

**Figure 2.**
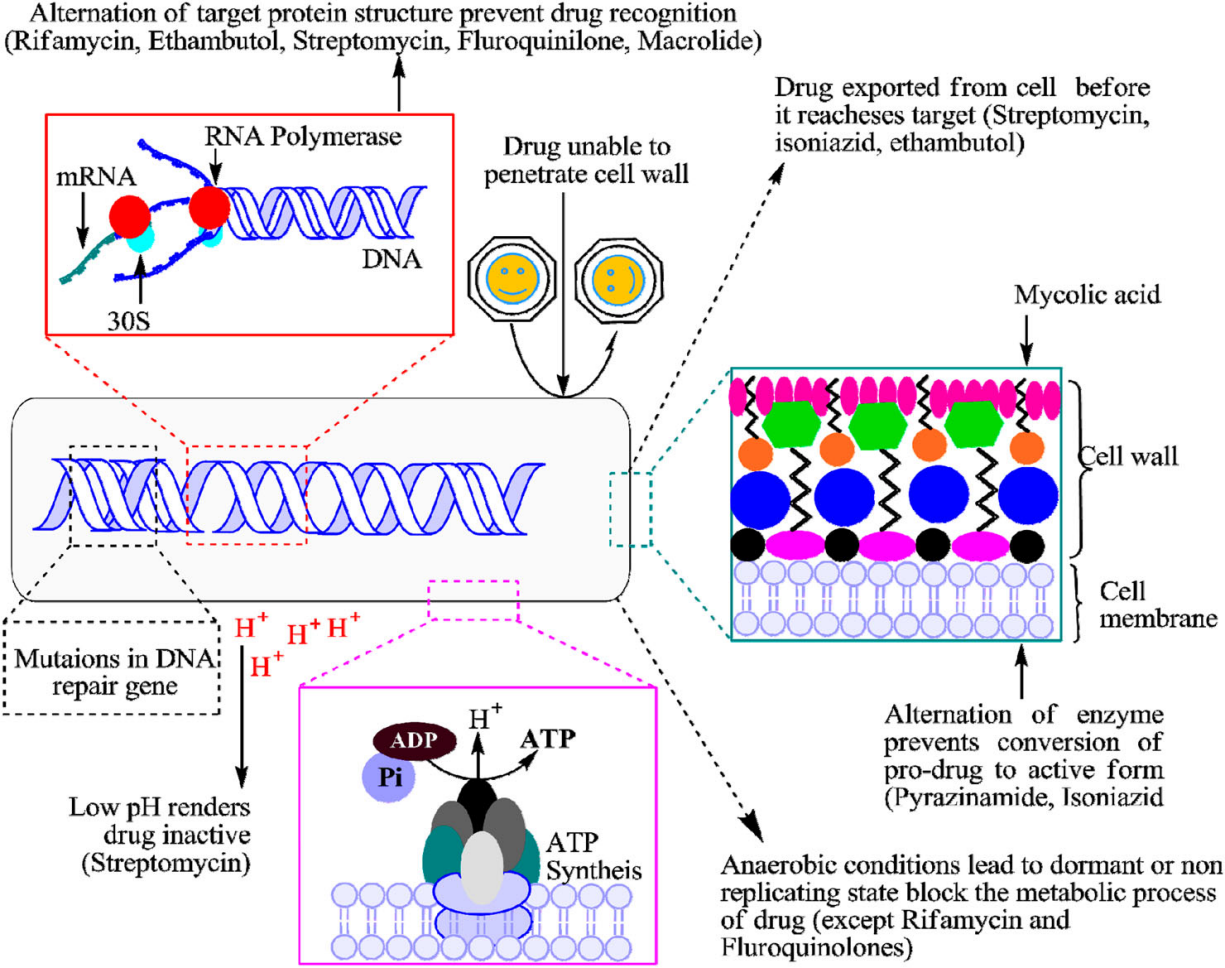
A schematic representation of the drug-resistant mechanism of mycobacterium tuberculosis.

Currently, drug-resistant Mtb strains spread as per Darwin evolution theory by overcoming the anti-TB regimens through adopting genetic mutations and other mechanisms along with selective environmental pressure [Figure 2]. Both long-term unremitting combination drug therapies and environmental conditions have created an evolution of drug-resistant Mtb strains which become resistant gradually against the existing anti-TB drugs [3,9,10]. Sometimes several environmental factors cause genetic modifications or mutations in the Mtb genome that not only reduce the effectiveness of the applied drug, but also provide the potential fitness for Mtb survival in any extreme condition [10]. This survival represents the fatal exposures to bactericidal antibiotics through radical-induced mutagenesis and promotes the multidrug-resistant phenotypes of Mtb. Sometimes, if this reactive oxygen and free-radicals fail to eradicate the mycobacterial cell, then bad promotion of cell mutagenesis and the rise of drug resistance [3,10].

Mostly, TB infection occurs through the engulfment of the bacterium by dendrite cells or alveolar macrophages, where bacilli avoid the killing mechanism and remain to reproduce evading phagosome-lysosome membrane fusion [11,12]. Complementary macrophages and other immune cells are usually contained in a specific site, known as granuloma. Particularly in the granuloma, Mtb bacilli are replicating vigorously along with non-replicating persistent/dormant form of Mtb in the specific site, which is prompted by the environmental circumstances, associated with anorexia/ hypoxia, nitric oxide production, and nutrient deprivation [11,13].

## Intrinsic resistance mechanisms in Mtb

The intrinsic mechanism of Mtb is resistance not only to the existing drugs but also to newly introduced drugs [Figure 2]. Broadly, the drug-resistant mechanisms of Mtb are divided into two categories, such as intrinsic and acquired for passively neutralizing the activity of applied anti-TB drugs/regimens [3,10]. Mtb attributed natural resistance mechanism against the macrolide class of antibiotics to low cell wall permeability through the expression of emr37. Particularly, emr37 belongs to such type of genetic factors that organize 23S rRNA-binding site through methylation [10,13]. Similarly, for natural resistance mechanism, the minimum inhibitory concentration (MIC) value of clarithromycin had decreased eight- to four-fold, by a permeability signalling barrier in the Mtb cell [10]. Using the intrinsic drug resistance, Mtb has continually developed the unfamiliar structure of cell wall that is related to low permeability against applying anti-TB chemotherapy [14].

In a study, the Mtb-Rv1698 has been directly associated with intrinsic resistance to hydrophilic antimycobacterial activities due to MspA genetic factors which is the porin-associated factor present in the Mtb cell wall that leads to lower the drug permeability [11,14]. The auto-regulator transcriptional activator WhiB7 controls the intrinsic drug resistance through the multidrug transporter tap, a transporter responsible for mycobacterial efflux against tetracycline or TET, STR and PAS, after binding with the somatic sigma factor, SigA [12,13]. Mainly, WhiB7, a small protein structure, contains 122 amino acids and an iron-sulphur group, which not only regulates the antibiotic resistance gene-like, eis, erm37 and tap, but also controls oxi-reductive reagents like dithiothreitol and diamide [12]. Subsequently, the eukaryotic-like protein kinase G (PknG), a type of virulence factor which is involved in the survival of Mtb in host-macrophages by regulating the redox-homeostatic system. Similarly, the nucleoid-associated transcription factor Lsr2 controls the oxygen level for the persistence of Mtb in host-cell through the expression of iniBAC, a promoter plays a crucial role during the inhibition of cell wall biosynthesis and EfpA, a transport protein associated with efflux mechanisms [14]. Additionally, the chromosomal protein Mar-regulon or MarA is also reducing the susceptibility of antibiotics like TET through regulating the MDR efflux pump. The overall antibiotic sensitivity and cellular redox status depend on the expression of NADH and reflux pump-associated transcription factors [12–15].

Thus, stress-responsive sigma factors (SigA, SigF), associated transcriptional proteins (MarA, SoxR and Rob) along with several specific oxidative stress, are significantly associated with the resistance of applied anti-TB drugs [3,11,16]_._ Several bioinformatic approaches have revealed the relationship between Mtb outer membrane proteins with intrinsic resistance against antibiotics in proteomic level [11,14]. However, apart from the permeability barriers of Mtb, physiological adaptations within the host can also manage antibiotic resistance [11,17]. Thus, understanding the intrinsic resistance is one of the major aspects in the Mtb drug development; because few drugs are available for treatment and could be a control in an iron hand of those gruesome Mtb strains by targeting intrinsic resistance-associated genetic factors [18,19].

## Acquired resistance mechanisms in Mtb

**Figure 3.**
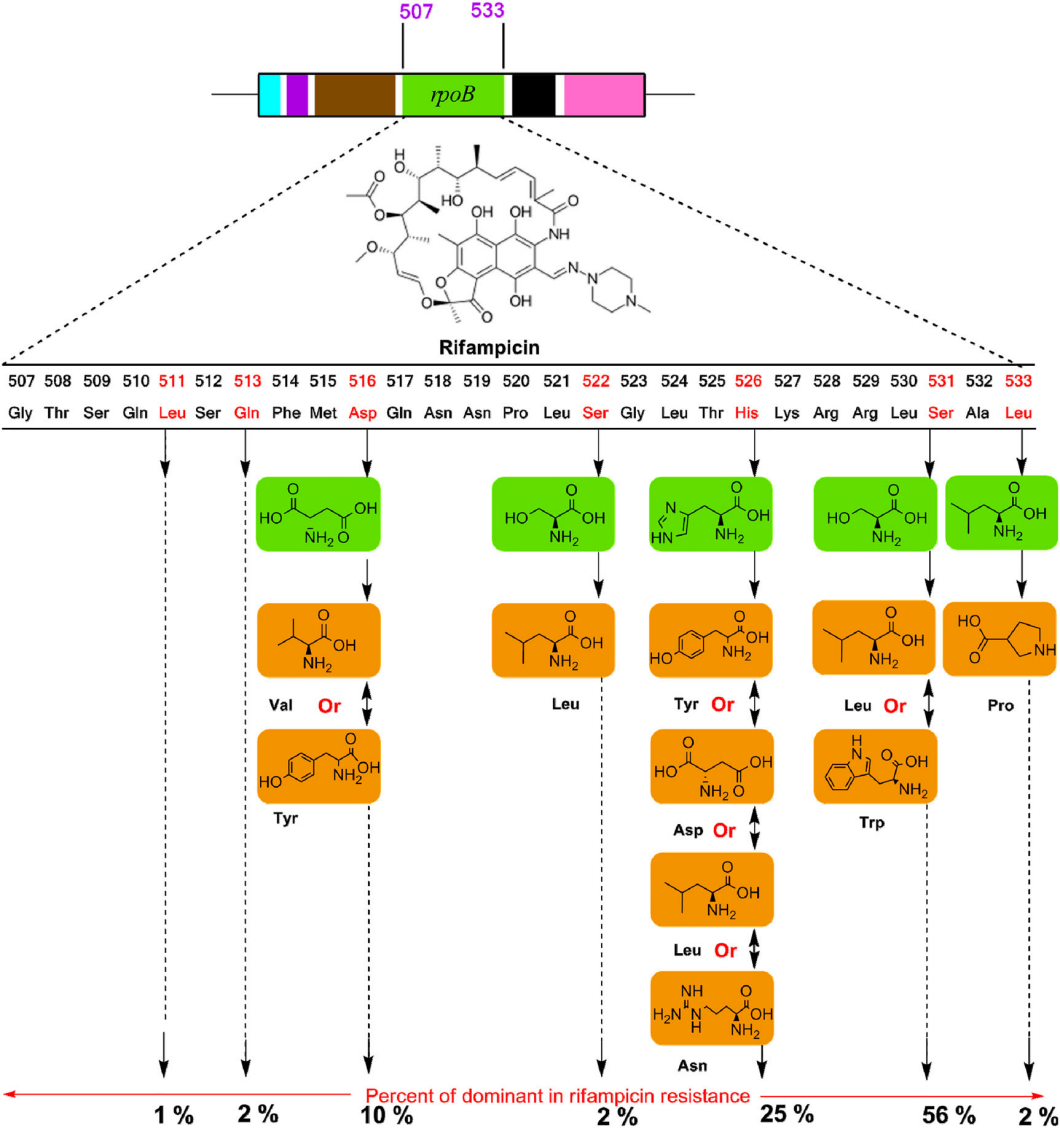
A schematic representation of genetic mutations associated with the drug-resistant mechanism of rifampicin targeting rpoB.

Acquired drug resistance is commonly facilitated by the horizontal transfer of mobile genetic elements such as plasmids and transposons. Acquired drug resistance occurs in two different ways, such as chromosomal or gene mutation mechanisms and extra-chromosomal or gene transfer mechanisms [3,10,19]. However, in Mtb, no such type of horizontal transfer of drug resistance genes has been reported, but most of them were found to be due to the origin of chromosomal mutations under selective pressure. Microorganisms also pay a physiological cost for drug resistance against anti-TB regimens. As a result, the rate of mutations in base pair is inversely genome size closely 0.0033 per replication in prokaryotes; however, in Mtb cases, most of the lead anti-TB regimens occur at 10^−9^ mutations per cell division [16,20]. Thus, the nature of drug selection is also directly related to the rate of mutation and that is the main ideal approach behind the combined formulation of anti-TB regimen in every single dose. The fitness cost of individual microorganisms depends on their growth and virulence transmission capacity from one host to another. For example, mutations on rpoB gene manage RIF-resistance in Mtb clinical isolates [Figure 3]; but sometimes the same isolates have less fitness cost *in vitro*. Thus, it may depend on mutations associated with some minor or major cost of the fitness of a strain [10]. Therefore, fitness cost depends on the specific resistance mutation and the genetic circumstances of the strain.

The fitness cost of various chromosomal mutations is directly proportional to antibiotic resistance, but there are limited data available on the resistance of drug and the fitness cost in Mtb. However, a chromosomal mutation is the most well-known mechanism of an antibiotic resistance [3,16,19]. Mutation in S315T on the catalase-peroxidase enzyme (katG) is the most pervasive mutation and nearly 40%–94% of resistance is associated with INH-resistant Mtb clinical isolates [3,10]. Mutations are capable of reducing the ability of katG to convert INH into iso-nicotinic acid, a precursor for the formation of the INH-NAD adduct in mycolic acid synthesis pathways [3,10]. However, RIF-resistant clinical isolates have been compared with their susceptible parental strain, four out of five strains with the mutation S531L and no fitness cost for this mutation. Similarly, mutations in the promoter region of enoylacyl carrier protein reductase (inhA) are associated with the over-expression of inhA which is the main cause of INH resistance. The frequently detected mutation at the position of C15T in inhA regulatory region is associated with INH resistance with MIC <1 μg/mL [21]. Only one plasmid is enough for carrying the resistance-encoded gene to overcome several drugs, the resistance of chloramphenicol, STR and TET are a suitable example [22]. Overall, antibiotics become ineffective in 10%–20% of the cases through chromosomal changes and ∼ 80% of cases of resistance by the extra-chromosomal process [22].

## Drug-resistant mechanisms of anti-TB drugs

Several newly introduced drugs were reportedly ineffective due to the emergence of MDR-TB, XDR-TB strains [3,4,23]. The current anti-TB regimens managed Mtb strains but not on a long-term basis as compared to STR. Cumulatively, environmental conditions, antibiotic pressure and chromosomal mutations play a major role in the development of drug resistant Mtb strain and transmission of those strains into a new host [10,13,16].

### Resistance mechanism of drug targeting cell wall, mycolic acid and folic acid synthesis

The Mtb cell wall comprises three covalently attached macromolecules, such as peptidoglycan, arabinogalactan and mycolic acid, known as “mycolyl-arabinogalactan-peptidoglycan” or mAGP complex [13,18,19]. The role of mycolic acid was well known and several anti-tubercular drugs were developed by targeting it and its associated enzyme in the biosynthesis, such as INH and EMB, used as a prodrug towards inhibition/alternation of mycolic acid biosynthesis targeting encoded enzymes, katG and inhA [10,22,24]. Particularly, INH makes a covalent adduct with the nicotinamide adenine dinucleotide (NAD) cofactor and that adduct complex acts as a tight-binding competitive inhibitor of inhA. However, mutations in the enzyme katG and inhA confer resistance to both EMB and INH [28]. S315T mutation of katG was considered as the universal mutation and nearly 40–94% INH resistance recorded in newer Mtb isolates [20]. Using advanced molecular tools, several other associated genetic factors related to cell wall biosynthesis (kasA, AhpC, niA, FadE24, ndh and FabG1) were explored in relation to anti-TB drug resistance [25,26]. Similarly, mutations in embB and embC cause resistance by Robinson transfers that are also associated with mycobacterial cell wall synthesis [27]. Recently, mutations in embB along with mutations in decaprenyl-phosphoryl-5-phosphoribose (DPPR) synthase gene (ubiA), are associated with a high level of EMB resistance. The cell wall targeting anti-TB drug, DCS, clofazimine, amoxicillin, meropenem, imipenem and thioacetazone have been recorded ineffective due to the resistance development by mutation with individual genetic factors, depicted in Tables 1–3 [28–53]. PAS is one of the most effective anti-TB drugs against multidrug-resistant tuberculosis since 1994 by targeting folic acid biosynthesis-associated enzyme, dihydropteroate synthase (DHPS) and iron metabolism inhibition. Nowadays, PAS has been ineffective due to mutation in the genetic factor, thyA gene (encoding with thymidylate synthase) and folC gene (dihydrofolate synthase or DHFS) [54]. Particularly, a mutation at Thr202Ala in thyA gene is most common for ∼ 37% of PAS-resistant strains [54,55]. Newly introduced two drugs, delamanid and pretomanid, have been more effective towards the inhibition of Mtb cell wall synthesis in the last couple of years; but recently, drug resistance against these two drugs have been reported across the glob [54–56].

**Table 1.** Genetic factors involved in first-line anti-TB drug resistance in *Mycobacterium tuberculosis*.

Drug (year)	Chemical class (activity type)	Inhibition target (associated action)	MIC (mg/L)	Genetic factor	Associated function	Drug resistance	Reference
Isoniazid (1952)	Isonicotinic acid (bactericidal)	Enoyl-(acyl-carrier-protein) reductase including, catalase peroxidase, NADH-dependent enoyl ACP, 3-Oxoacy ACP, *β*-Ketoacyl ACP) (inhibition of cell wall synthesis)	0.02-0.2	katG	Intracellular survival	Modification overexpression of drug target due to mutations and altered efflux pump activity and pro-drug conversion	[28,29]
				inhA	Mycolic acid biosynthesis		
				kasA	Fatty acid biosynthesis		
				AhpC	Defence from oxidative stress		
				niA	Associated with efflux pump		
				FadE24	Degradation lipid and fatty acid		
				ndh	Electron transference from NADH to the respiratory chain		
				FabG1	Fatty acid biosynthesis pathway		
Rifampicin (1963)	Rifamycin (bactericidal)	RNA polymerase, *β*-subunit (inhibition of RNA synthesis)	0.05-1.0	rpoB rpoA rpoC	Catalyze the transcription for DNA into RNA synthesis	Modification in drug target due to mutations	[30,31]
Ethambutol (1961)	Ethylenediamine (bacteriostatic)	Arabinosyl transferase (inhibition of arabinogalactan synthesis)	1–5	emb A, B and C	Associated with biosynthesis of the mycobacterial cell wall	Change and overexpression of drug target; and altered efflux pump activity	[29,32]
				embR	embCAB operon synthesis regulator		
				rmLD	dTDP-L-rhamnose biosynthesis		
				iniA	Associated with efflux pump		
Pyrazinamide (1952)	Pyrazine (bacteriostatic/bactericidal)	Pyrazinamidase; ribosomal protein 1 30S ribosomal subunit, cytoplasm	16–100	pncA	To converts amides into acid	Abolition of pro-drug conversion mechanism	[33,34]
				rpsA	Translate mRNA with a shine-dalgarno purine-rich sequence		
				panD	Pantothenate biosynthesis		
				clpC1	Protein degradation, hydrolyses proteins in the presence of ATP		
				gpsI	Involved in mRNA degradation		
Streptomycin (1944)*	Aminoglycoside (bacteriostatic)	30S, 16S ribosomal protein and 7-methyl guanosine methyl-transferase (inhibition of arabinoga-lactan and protein synthesis)	2–8	rpsL	Translation initiation step	Variation in drug target binding site due to mutations	[35,36]
				rrs	Synthesis of stable RNAs		
				gidB	Probable glucose-inhibited division protein B		

*, injectable.

**Table 2.** Genetic factors involved in second-line anti-TB drug resistance in *Mycobacterium tuberculosis*.

Drug (year)	Chemical class (activity type)	Inhibition target (associated action)	MIC (mg/L)	Genetic factor	Associated function	Drug resistance	Reference
Moxifloxacin (1996) Gatifloxacin (1999)	Quinolones/8-methoxyfluroquinolone (bactericidal)	DNA gyrase and DNA topoisomerase (Inhibits DNA synthesis)	0.5–2.5	gyrA, gyrB	Negatively supercoils closed circular double-stranded DNA	Alteration of drug target due to mutation	[29,37]
Kanamycin* (1957) Amikacin* (1972)	Amino-glycosides (bactericidal)	Inhibition of RNA-dependent synthesis by binding to 30S subunit (inhibition of protein synthesis)	2–8	rrs	Synthesis of stable RNAs	Mutations on 16S rRNA and overexpression machines	[36,38]
				eis	Acetylation, intracellular survival		
				whiB7	Associated with transcription		
				tlyA	Methylates 16S and 23S rRNA		
Capreomycin* (1963)	Cyclic polypeptide (bactericidal)	Inhibition of 50S subunit (inhibition of protein synthesis)	2–4	rrs	Synthesis of stable RNAs	Mutation alteration drug target	[39,40]
				tlyA	Methylates 16S and 23S rRNA		
				eis	Acetylation, intracellular survival		
Ethionamide (1956)	Isoconitic acid derivative (bacteriostatic)	Inhibition of mycolic acid synthesis by binding to the ACP reductase InhA (disrupts cell wall biosynthesis)	2.5–25	ethA	Activates the pro-drug ethionamide	alteration and over-expression drug target due mutation	[40,42]
				ethR	Regulates transcriptional repressor protein EthR		
				KasA	Involved in fatty acid biosynthesis		
				inhA	Mycolic acid biosynthesis		
				inhA pro.	Regulation of expression of inhA		
*Para*-amino salicylic acid (1946)	*Para*-amino salicylic acid (bacteriostatic)	Dihydropteroate synthase (inhibits folate and thymine nucleotide metabolism biosynthesis)	1–8	thyA	Deoxyribo-nucleotide biosynthesis	Removal of pro-drug conversion procedure	[36,42]
				folC	Regulate folates to polyglutamate conversion		
				dfrA	De novo glycine and purine synthesis		
				ribD	Involved in riboflavin biosynthesis		
Cycloserine (1955)	Serine derivative (bacteriostatic)	Inhibition of peptide-glycan synthesis by blocking d-alanine racemase enzyme (inhibition of cell wall synthesis)	25–30	alr	Associated with d-alanine required for cell wall biosynthesis	Overexpression of *resistance gene*	[43,44]
				ddl	Involved in cell wall formation		
				Ald	Associated with cell wall synthesis		
				cycA	Transport across the cytoplasmic membrane		

*, injectable.

**Table 3.** Genetic factors involved in third-line anti-TB drug resistance in *Mycobacterium tuberculosis*.

Drug (year)	Chemical class (activity type)	Inhibition target (associated action)	MIC (mg/mL)	Genetic factor	Associated function	Drug resistance	Reference
Clofazimine (1954)	Iminophenazine derivative (*bacteriostatic*)	Produces reactive oxygen, inhibits energy production, potassium transporter (inhibition of mycobacterial growth targeting mycobacterial DNA)	0.1–1.2	rv0678	Transcription repressor for efflux pump MmpL5	Upregulation of MmpL5, efflux pump due to mutation	[45,46]
				rv1979c	Role in the transportation of amino acid		
				rv2535c	Associated/encodes a putative peptidase PepQ.		
				ndh	Associated with oxidation and reduction reaction		
				pepQ	Possibly hydrolyse peptides		
Bedaquiline (2012)	Quinoline (bactericidal/ bacteriostatic)	Inhibits the adenosine 5'-triphosphate synthase (inhibition of ATP synthase)	0.06–1	rv0678	Transcription of efflux pump MmpL5	Mutations on binding site and co-infection	[47–48]
				atpE	Encodes the c part of the F0 subunit of the ATP synthase		
				pepQ	Possibly hydrolyses peptides		
Delamanid (2014) Pretomanid (2020)	Nitroimidazole (bactericidal)	Obstructs the synthesis of mycolic acid	0.006–0.24/0.015–0.25	fgd1	Catalyzes oxidation of glucose-6-phosphate to 6-phosphogluconolactone	Mutations on reductive activating gene	[49–51]
				fbiC	Participates in a portion of the F420 biosynthetic pathway		
				fbiA	Required for coenzyme F420 production from FO		
				fbiB	Required for coenzyme F420 production from FO		
				ddn	Converts bicyclic nitroimidazole drug candidate pa-824–3 metabolites		
Linezolid (2000)	Oxazolidinone (*bactericidal*)	50S, 23S ribosomal subunit (inhibition of protein synthesis)	0.25–0.5	rplC	Formation of ribosomal peptidyl-transferase	Mutation in 50S ribosomal L3 protein	[52,53]
				rrl	Formation of stable RNAs		

Several chemicals have been designed to target the aminoacyl-tRNA synthetases (AARS)-associated enzymes which are essential enzymes required for bacterial protein synthesis [57]. Moreover, some unique lipids are present in the Mtb cell envelope and act as a permeability barrier to use anti-TB drugs. As a result the lipid barrier plays an essential role in Mtb drug resistance [58]. The structurally distinct mycobacterial lipids, derived from malonyl coenzyme A (CoA) are assembled with acyl-CoA carboxylases (ACC) subunits, AccA1 to AccA3, AccD1 to AccD6, AccE5. Moreover, ACC is an essential factor for fatty acids, mycolates and lipid synthesis of Mtb along with biotin (vitamin H or vitamin B7), as the cofactor in the post-translational process [59–62]. Thus, *de novo* biotin biosynthesis or biotin-ACC ligation inhibition is a new target for Mtb inhibition.

### Resistance mechanism of drug targeting ATP, DNA, RNA and protein synthesis

The pandemic Mtb-resistant traits arising through evolution, antibiotic pressure and other means which deliver this mutant feature for their survival in a new environment [20,21,36]. Due to a point mutation, the applied drugs cannot bind the proper sites of a target and as a result decrease the potency [10,13,14,17]. The central dogma pathways, such as DNA, RNA and protein synthesis, are encoded nodal enzymes and precursors to achieve the required integration for surviving in an environment or a host body. Thus, several anti-TB drugs are developed targeting ATP, DNA and protein synthesis, because these are crucial to produce energy for the survival of Mtb [3,10,16]. Diarylquinoline and imidazopyridine amide class of antibiotics/drugs are targeting for the inhibition of ATP synthesis of Mtb, PAS inhibits DNA precursor, and fluoroquinolone class of antibiotics inhibits DNA gyrase of Mtb [48]. RIF inhibits RNA synthesis, while oxazolidinones (linezolid), aminoglycosides, macrolides, and cyclic peptides inhibit protein synthesis, among them linezolid is the potential orally existing drug targeting Mtb protein synthesis. Mutated rpoB encourages a conformational change towards the binding affinity of RIF at β-subunit of the RNA polymerase, and the drug became inactive without proper binding to the exact target site [30,31; Figure 3].

## Future prospective of drug resistance and drug development

For a long time, there is a duel between humans and bacteria for surviving from each other. The development of antibiotics changed the whole thing for several decades. Nowadays, Mtb produce more unique genetic factors and develop quorum sensing bio-film mechanism to counter-attack against the new anti-TB drug [14–17]. Modern mutants can regain fitness for enhancing transmissibility of virulence from adopting a resistance mechanism from the existing mutant strains with a suitable environmental pressure [20,23,24]. In the past, several MDR epidemic analyses confirmed that evolution of regulatory systems by an antibiotic during the transition of Mtb from a dormant state to active growth and increased the phenotypic drug tolerance of latent TB.

In the current perspective, drug-resistant TB is associated with genetic mutations on the Mtb target enzyme [27,34,44,47,55]. The resistance factor related to variations is broadly divided into two types i.e. cellular mechanisms connected with mismatch renovation, DNA polymorphisms error, translational inaccuracy, etc., and external mechanisms connected with stress factor, host-environment, pollution, quality of diagnosis, etc. [17–19]. Additionally, inadequate hospital facility, inappropriate guidance, long-term hospital expenses, lack of strict national antibiotic policy, awareness, illiteracy and poverty are some of the associated social-economic factors in drug-resistant TB [63,64]. In short, the non-cooperation of TB patients and costs are directly affecting to achieve the complete cure. Thus, TB is one of the primary causes of death in the Asian population and the more common cause of adult mortality than HIV, malaria and other tropical infectious diseases [1,4,65]. The development of co-infection of TB with HIV has lead to the increase in MDR-TB, MDR-TB and XDR-TB strains [1,2]. Mostly, rates of TB incidence tripled in African countries with high HIV prevalence. Approximately 13% of HIV-TB cases were recorded from global TB cases; among them 39% HIV-TB from Africa [1,2]. As a result, complex resistance profiles with intersecting toxicity and a substantial amount of drug consumption obstruct towards the control of co-infected TB.

Continuously approved anti-TB drugs bedaquiline, delamanid and pretomanid in a small duration encouraging the drug development performance. But we need to develop more and more to control millions of TB-infected people, globally [4,8,48–50]. Approximately, ∼ 265 billion dollars for the control of TB as for the development of a new diagnosis and treatment in the medical research, globally, but the output is only 0.25% [4,8,66]. From WHO report, there are several anti-TB agents in the pipeline and hope for a discovery in the recent future. According to the current reports, most tubercular agents contain nitrogen heterocyclic and pyridine and pyrazine derivatives similar to the existing anti-TB drug, INH, PZA, etc. Precisely, nitrogen heterocyclic containing nucleus is an essential chemical scaffold for a future anti-TB drug with changed physicochemical, metabolic and pharmacokinetic properties, through the addition of a potential side chain molecule [67,68].

**Figure 4.**
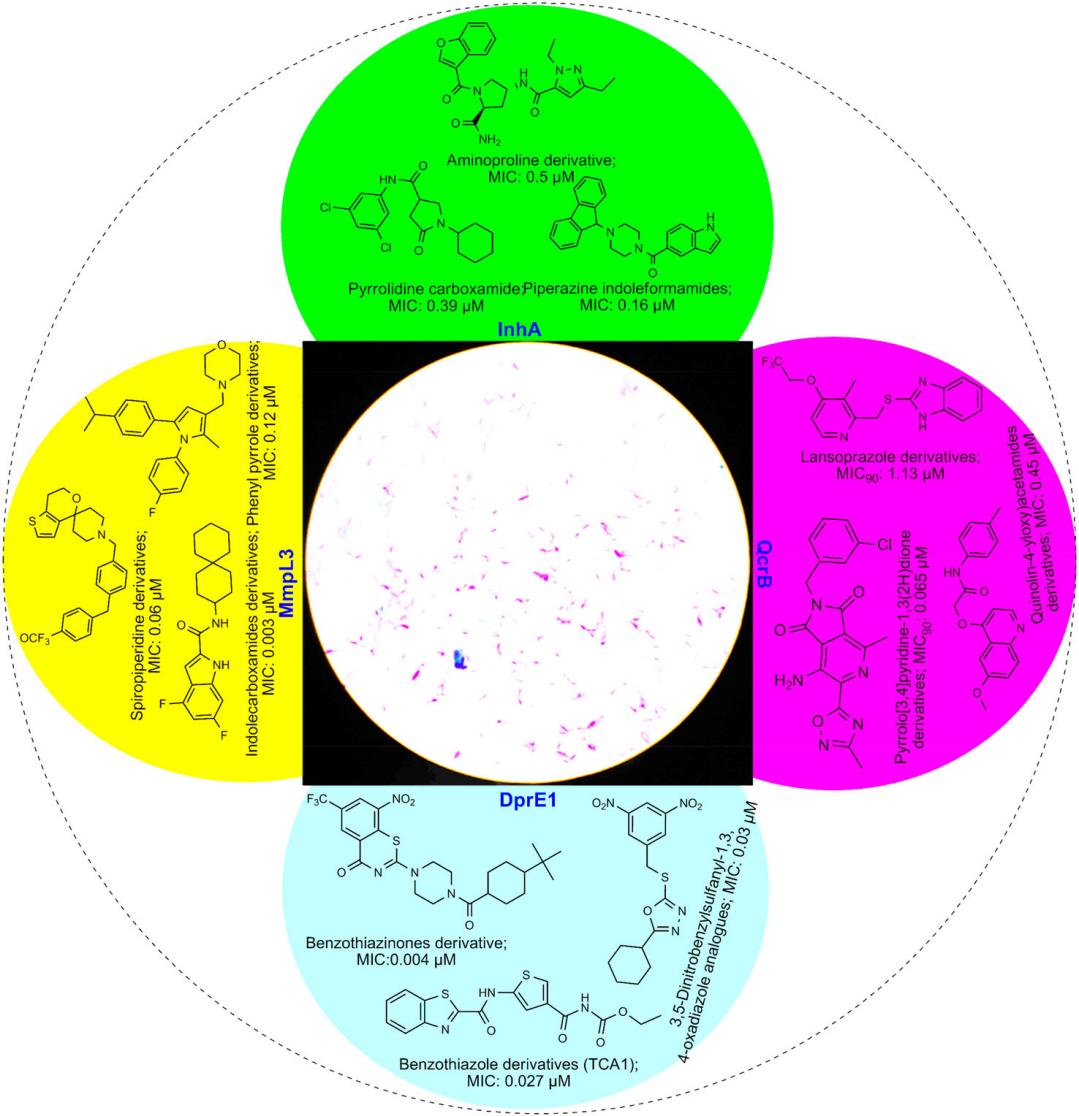
Newly identified anti-TB drug development targets and pipeline drugs.

The excellent progress screening procedure with possible adopted techniques in medicine and molecular mechanisms is expected to the development of the effective anti-TB drug in recent future [Figure 4]. Besides, towards searching for a new anti-TB regimen, natural phyto-metabolites (secondary plant metabolites) and phyco-chemical (cyanobacterial compound) have high attention due to their potential reported towards promoting health and reducing disease burden [5–8]: terpenoid class of phytochemicals, mono-O-methyl curcumin isoxazole from well-known Indian medicinal plant, *Curcuma longa* with the MIC value, 00019 mg/mL and abietane from a Chinese sub-species, *Plectranthus grandidentatus*, with the MIC value 0.00039 mg/mL, the quinone class of phyto-compound, plumbagin from *Diospyros anisandra*, with the MIC value, 0.0015–0.0033 mg/mL and 7-methyljuglone from the South African medicinal shrub *Euclea natalensis* with the MIC value 00057 mg/mL, an organic heterocyclic compound, calanolide A from the Malaysian sub-species, *Calophyllum lanigerum*, with the MIC value, 0.0031–0.016 mg/mL, the alkaloid class of constitution, dihydro-β-agarofuran sesquiterpene from *Celastrus vulcanicola,* with 0.0062 mg/mL [69–72].

Similarly, several unique bioactive marine alkaloid class of products, such as Ecteinascidin 770 from the sea squirt, *Ecteinascidia thurstoni* with the MIC value 0.00013 mg/mL, Hirustellone A-D from the fungi, *Hirustella nivea* BCC2594 with the MIC value 0.00078 mg/mL, 8-Hydroxymanzamine J from marine sponge *Acanthostrongylophora* sp., with the MIC value 0.00040 mg/mL were recorded against Mtb [73,74]. Additionally, the phenolic class of chemical nocardithiocin from marine bacterium *Nocardia pseudo brasiliensis* with the MIC value 0.00002–0.00115 mg/mL, Trichoderin A from marine fungi *Trichoderma* sp., with the MIC value, 0.00012 mg/mL, Lariatin A from marine bacterium *Rhodococcus jostii* with the MIC value 0.00039 mg/mL were recorded as most preferable anti-TB candidates [75–77].

In comparison, plant chemicals are ideal drug candidates than the marine chemicals in the host-toxicity point of view; however, the drug success rate is higher in marine products than plant products [5,6]. Thus, an ideal drug candidate should manage both activity and toxicity with pharmacokinetic properties for more success in drug validation modules. Strategically, several advanced chemical conjugation/drug modification through medicinal chemistry protocol and nanoparticle/nanocarrier formulations has been adopted for the utilization of more natural products in anti-TB drug development [78–80]. Nevertheless, long-term investment, administrative commitment, interior geographical support and scientific endeavour will be playing the crucial factors for a sustainable TB-free society.

## Conclusion

The prevalence rate of Mtb and resistance mechanisms against ongoing therapy is a serious global concern. The absence of potential drug candidates and patient awareness along with unhygienic practices are the prime reasons for rapid emergence of MDR, TDR, XDR Mtb strains. Understanding and analysis of compensatory evolution, clonal interference, declined cell wall permeability, overexpression of efflux pumps, target modification and target mimicry modulates are the key factors associated with drug resistance. The powerful molecular mechanism with complex pathway information of drug resistance of Mtb immensely requires counter-attacking towards the development of new anti-TB regimens. Hopefully, the combination of new genomics knowledge of drug-resistant mechanisms in Mtb would give a new direction for combination drug discovery and tremendous support for the production of highly effective anti-TB drugs.
